# Stromal PDGFR-beta expression is a prognostic factor in high-grade serous ovarian cancer patients but is it also predictive for response to antiangiogenic treatment?

**DOI:** 10.1007/s00432-025-06090-4

**Published:** 2025-01-24

**Authors:** Annabelle Volk, Charlotte von Bülow, Aysche Stern, Ronald Simon, Eike Burandt, Guido Sauter, Barbara Schmalfeldt, Leticia Oliveira-Ferrer

**Affiliations:** 1 Department of Gynecology, University Medical Center Hamburg-Eppendorf, Martinistrasse 52, 20246 Hamburg, Germany; 2 Department of Pathology, University Medical Center Hamburg-Eppendorf, Hamburg, Germany; 3 Department of Internal Medicine, Southwest Healthcare, Temecula, USA

**Keywords:** Ovarian cancer, PDGFR-beta, VEGFR-2, Bevacizumab, Prognostic biomarker

## Abstract

**Objective:**

In advanced ovarian cancer, the majority of patients receive anti-angiogenic treatment with bevacizumab. However, its use is often associated with severe side effects, and not all patients benefit from the therapy. Currently, there are no reliable biomarkers to predict response to treatment. Given their role as key regulators of angiogenesis, platelet-derived growth factor receptor-beta (PDGFR-beta) and vascular endothelial growth factor receptor-2 (VEGFR-2) are promising candidates for predictive biomarkers. This study evaluates their potential.

**Methods:**

PDGFR-beta and VEGFR-2 expression was evaluated using immunohistochemistry in a tissue microarray assay including 391 ovarian tissue samples. Correlation analyses with clinical and histopathological parameters were performed in a homogeneous cohort of 199 high grade serous ovarian cancer samples (HGSOC).

**Results:**

In HGSOC, strong stromal PDGFR-beta expression was associated with significantly shorter overall survival compared to weak/moderate expression. The impact of stromal PDGFR-beta expression on patient survival was however not restricted to the subgroup of patients receiving therapy with bevacizumab, and therefore cannot be considered as a predictive factor. For VEGFR-2, no or weak protein expression was found in the majority of the tumor samples. Survival analyses showed a more favorable prognosis with no or weak VEGFR-2 expression.

**Conclusions:**

High stromal expression levels of PDGFR-beta correlate with shorter overall survival in HGSOC. Thus, stromal PDGFR-beta might serve as a prognostic biomarker. No predictive effect in response to bevacizumab therapy could be attributed.

**Supplementary Information:**

The online version contains supplementary material available at 10.1007/s00432-025-06090-4.

## Introduction

Ovarian cancer is the second leading cause of death from gynecological cancer (Lheureux et al. 2019). It is characterized by an overall poor prognosis with a 5-year survival rate of less than 50% (Torre et al. 2018). High mortality is associated with advanced stages of disease at first diagnosis (Cabasag et al. 2022).

Treatment of choice consists of cytoreductive surgery followed by adjuvant platinum-based chemotherapy (Colombo et al. 2019). Studies have shown that the addition of bevacizumab (7,5–15 mg/kg every three weeks) to chemotherapy followed by a 10-month-maintenance therapy with bevacizumab only, lead to a significant increase in progression-free survival by 4 months (Burger et al. 2011; Garcia and Singh 2013).

Bevacizumab is a humanized anti-VEGF antibody that targets VEGF-A, a signal molecule with impact on the regulation of tumor angiogenesis (Presta et al. 1997). It is approved for advanced stage epithelial ovarian cancer FIGO IIIB-IV in primary and recurrent situation. Since ovarian cancer is mainly diagnosed in advanced stages of disease, the majority of patients receive treatment with bevacizumab. However, it is questionable to use clinical stage as the only selection criterion. It has been shown that anti-angiogenetic treatment has no impact on overall survival (Burger et al. 2011). Considering significant side effects and costs of therapy, identification of patients who are most likely to benefit from anti-angiogenic treatment with bevacizumab is of great value (Monk et al. 2016). We have previously shown that the angiogenic growth factor angiopoietin-2 is a potential predictive marker for response to therapy with bevacizumab (Volk et al. 2023).

Platelet-derived growth factor (PDGF) and their corresponding tyrosine kinase receptors (PDGFRs) are known to be involved in angiogenesis and mediate pathogenesis of various tumor types (Berndsen et al. 2019). When PDGF binds to PDGFR-alpha and -beta, the two receptor isoforms initiate signaling cascades leading to cell proliferation, cell survival, cell differentiation, chemotaxis and migration (Andrae et al. 2008).

Vascular endothelial growth factor (VEGF) is a signaling molecule also known to be involved in angiogenesis, as well as proliferation and migration during development of endothelial cells (Ferrara et al. 2003). VEGF initiates its role as a key regulator by binding to VEGF-receptor (VEGFR) −1 and −2, of which VEGFR-2 is the primary mediator for the physiological effects in angiogenesis (Mabeta and Steenkamp 2022). Higher expression of VEGFR-2 in tumor cells and its microenvironment compared to normal tissues have been observed (Wang et al. 2020).

In our previous work, significant overexpression of PDGFR-beta and VEGFR-2 was found in platinum-resistant tumors. A prognostic value could be ascribed to both proteins, as the analyses showed a significant correlation between higher levels of PDGFR-beta and VEGFR-2 and shorter overall survival (Avril et al. 2017).

Based on these findings and the knowledge that both proteins play a key role in angiogenesis, we carried out expression analyses with an even larger cohort (n = 391) in a tissue microarray assay and examined, among other things, the correlation between expression and response to anti-angiogenic treatment with bevacizumab.

## Materials and methods

### Patients

A total of 391 patients with ovarian tumors, including epithelial ovarian cancer (EOC), Mixed Müllerian tumor (MMT), borderline ovarian tumors (BOT), teratoma and healthy tissue treated at the University Medical Center Hamburg-Eppendorf between 2009 and 2017 were included in this study. All patients gave written informed consent to obtain biomaterial and access their clinical records. The study has been approved by the ethics committee (Ethik-Kommission der Ärztekammer Hamburg, no. 190504) in advance. Patients’ data was tracked from date of first diagnosis until 2021.

### Tissue microarray (TMA)

The TMA used in this study has been described before (Rico et al. 2021). In brief, the TMA was made from 603 ovarian cancers diagnosed at the University Medical Center Hamburg-Eppendorf between 2009 and 2018. For this study, only the subset of 391 tumors was analyzed for which detailed clinical data was available. This included 265 serous, 30 mucinous, 24 endometrioid, and 20 clear cell carcinomas, 13 malignant mixed Mullerian tumors, and 39 other diagnoses (Table 1). TMA construction was done as previously described (Mirlacher and Simon 2010). Clinical and histo-pathological data of the overall cohort is listed in Table 1. The use of archived remnants of diagnostic tissues for manufacturing of tissue microarrays and their analysis for research purpose as well as patient data analysis has been approved by local laws (HmbKHG, §12) and by the local ethics committee (Ethics Commission Hamburg, WF-049/09). All work has been carried out in compliance with the Helsinki Declaration.

**Table 1:** Patients characteristics

		Overall cohort (*n* = 391)	HGSOC (*n* = 205)
		*n* (%)	*n* (%)
Diagnosis	EOC	349 (89.3)	205 (100.0)
	MMT	13 (3.3)	
	BOT	12 (3.1)	
	Teratoma	1 (0.3)	
	Healthy tissue	6 (1.5)	
	Not specified	10 (2.5)	
FIGO stage	I	61 (15.6)	6 (2.9)
	II	23 (5.9)	9 (4.4)
	III	218 (55.7)	144 (70.2)
	IV	73 (18.7)	41 (20.0)
	Not specified	16 (4.1)	5 (2.4)
Grading	G1	38 (9.7)	
	G2	51 (13.0)	
	G3	254 (65.0)	205 (100.0)
	Not specified	48 (12.3)	
pT	pT1a	29 (7.4)	1 (0.5)
	pT1b	5 (1.3)	3 (1.5)
	pT1c	37 (9.5)	6 (2.9)
	pT2a	5 (1.3)	4 (2.0)
	pT2b	21 (5.4)	7 (3.4)
	pT2c	9 (2.3)	6 (2.9)
	pT3	3 (0.8)	2 (1.0)
	pT3a	13 (3.3)	6 (2.9)
	pT3b	47 (12.0)	32 (15.6)
	pT3c	190 (48.6)	135 (65.9)
	Not specified	32 (8.1)	3 (1.5)
Nodal	N0	116 (29.7)	49 (23.9)
involvement	N1	176 (45.0)	119 (58.0)
	Not specified	99 (25.3)	37 (18.0)
Histological	Serous/serous-papillary	265 (67.8)	205 (100.0)
type (EOC)	High-grade serous	205 (52.4)	
	Low-grade serous	15 (3.8)	
	Endometrioid	24 (6.1)	
	Clear cell	20 (5.1)	
	Mucinous	30 (7.7)	
	Not specified	10 (2.6)	

Residual tumor no residual tumor152 (38.9)91 (44.4) after surgery < 1 cm53 (13.6)37 (18.0) > 1 cm26 (6.6)20 (9.8) not specified160 (40.9)57 (27.8)

Chemotherapy platinum-based159 (40.6)74 (36.1) platinum-based + bevacizumab166 (42.5)131 (63.9) no chemotherapy1 (0.3) not specified65 (16.6)

Survival alive258 (66.0)118 (57.6) deceased133 (34.0)87 (42.4)

Tumor yes201 (51.4)137 (66.8) recurrence no176 (45.0)66 (32.2) unknown14 (3.6)2 (1.0)

Overall survival median24 months29 months range0-136 months0-136 months

Progression free median21 months21 months survival range2-67 months4-67 months

### Immunohistochemistry (IHC)

Freshly prepared TMA sections were immunostained on one day in one experiment. Slides were deparaffinized and exposed to heat-induced antigen retrieval for 5 min in an autoclave at 121 °C in pH 9.0 Target Retrieveal Solution (Agilent, Santa Clara, CA, USA). Primary antibody specific against PDGFR-beta protein (rabbit monoclonal, Cell Signaling Technology, Danvers, MA, USA, clone C82A3) was applied at 37 °C for 60 min at a dilution of 1:150. Primary antibody specific against VEGFR-2 protein (rabbit monoclonal, Cell Signaling Technology, Danvers, MA, USA clone 55B11) was applied at 37 °C for 60 min at a dilution of 1:150. Bound antibody was then visualized using the EnVision Kit (Dako, Glostrup, Denmark) Itaccording to the manufacturer’s instructions. Immunohistochemical evaluation was performed by two independent investigators.

Staining scores ranged from 0 to 3. Score 0 indicates complete absence of detectable staining (negative), while score 3 is defined as intense dark brownish staining of cell membranes and the cytoplasm (strong). Scores 1 and 2 indicate staining intensities between scores 0 and scores 3, with score 1 meaning that the staining is clearly visible but of weak intensity, while score 2 indicates moderate staining not reaching the intensity of score 3. Representative images of the scores are shown in supplementary Fig. 1 for PDGFR-beta and in supplementary Fig. 2 for VEGFR-2. For statistical analyses, a homogenous cohort including only tissue from epithelial ovarian cancer with serous and serous papillary histological subtype (n = 265) was considered.

### Statistical analyses

For statistical analyses SPSS Version 27.0 was used. Protein expression was correlated with clinical- and pathological parameters by Pearson’s chi-squared test. Using Kaplan–Meier method, survival curves were created and differences between curves were tested using Log-rank test. Probability values less than 0.05 were regarded as statistically significant.

## Results

### Tissue microarray analysis on tissue samples of ovarian cancer patients

Initially, tissue samples of 391 patients included in a TMA were analyzed. Samples included tissue from EOC, MMT, BOT, teratoma and healthy tissue. Staining scores ranged from 0 to 3. With weak staining defined as 1, moderate staining as 2 and strong staining as 3, while 0 was used in case of no staining. Clinical and histo-/pathological data of the overall cohort is listed in Table 1. Due to the small number of tissue samples of MMT, BOT, teratoma and healthy tissue, a homogenous subcohort including only tissue from epithelial ovarian cancer with serous and serous papillary histological subtype (n = 265) was considered for all statistical analyses presented in the next sections.

### PDGFR-beta expression analysis on tissue samples of ovarian cancer patients

**Fig. 1:**
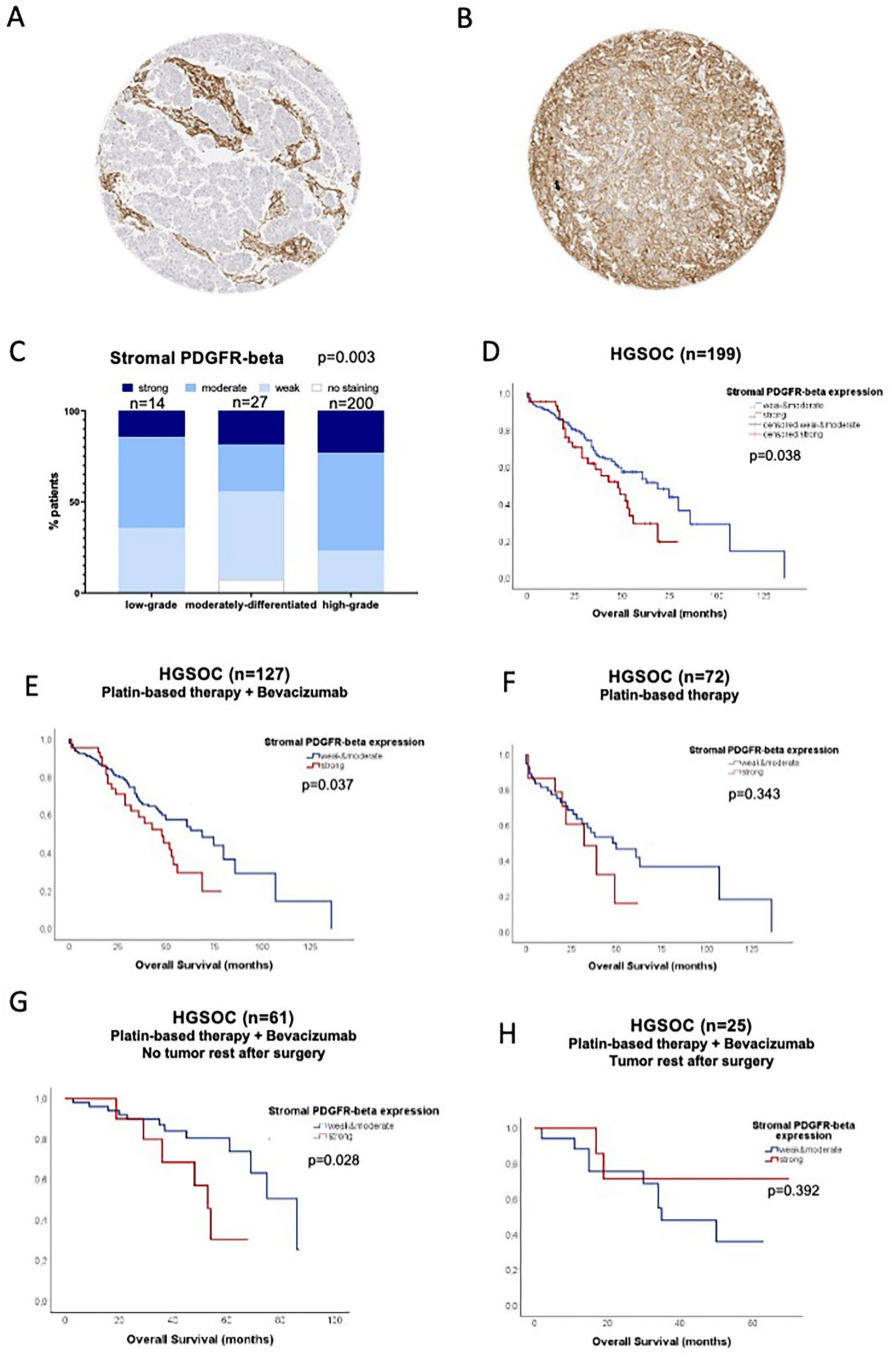
Stromal PDGFR-beta expression analysis on tissue samples of serous ovarian cancer patients. **A**–**B** PDGFR-beta immunostaining in serous ovarian carcinoma: **A** Absent PDGFR-beta staining in the tumor cells, while the surrounding stroma shows strong expression. **B** Strong PDGFR-beta staining in the tumor and the stroma. **C** Stromal PDGFR-beta expression correlates significantly with histopathological grading (*p* = 0.003). **D** In high-grade serous ovarian cancer strong PDGFR-beta expression correlates significantly with shorter overall survival (*p* = 0.038). **E** After dividing the cohort with respect to therapy regimens, a significant association is only seen in patients treated with bevacizumab therapy (*p* = 0.037). **F** In patients that did not receive bevacizumab therapy, PDGFR-beta expression levels have no significant impact on overall survival (*p* = 0.343). **G** Moreover, in the cohort of ovarian cancer patients with no residual disease after cytoreductive surgery, treated with bevacizumab, high PDGFR-beta expression correlates significantly with shorter overall survival (*p* = 0.028). **H** In patients with residual disease after cytoreductive surgery, that received bevacizumab therapy, PDGFR-beta expression levels have no significant impact on overall survival (*p* = 0.392).For all Kaplan–Meier analyzes he cohort was divided into two groups according to the intensity of stromal PDGFR-beta staining (weak to moderate staining vs. strong staining)

PDGFR-beta expression was detected at different intensities in stromal tissue. Staining was weak in 68 (25.7%), moderate in 133 (50.2%) and strong in 53 (20.0%) tumors. 3 (1.1%) tumor samples showed no staining and 8 (3.0%) tumors were not analyzable. Regarding the PDGF-beta staining on tumor cells, the majority of 208 (78.5%) samples showed no staining and 25 (9.4%) tumors were not analyzable. Positive staining was noted in only 32 (12.1%) samples, out of which 24 (9,1%) samples were stained weakly, 3 (1.1%) moderately and 5 (1.9%) strongly. Representative images of samples with different levels of stain intensity are shown in Fig. 1A–B. Due to the small number of samples showing PDGFR-beta tumor cell staining, we focused on the relevance of stromal PDGF-beta for further analysis.

### Stromal PDGFR-beta expression correlates significantly with histopathological grading in serous ovarian cancer

Statistical analyses revealed no significant association between stromal PDGFR-beta expression levels and tumor stage, FIGO, nodal involvement, distant metastasis or postoperative tumor residual (Table 2). However, stromal PDGFR-beta staining intensity correlated significantly with grading (p = 0.003, Fig. 1C). Here, higher levels of stromal PDGR-beta were found in high-grade serous carcinomas (HGSOC) compared to low-grade and intermediate-grade (G2) serous carcinomas.

**Table 2:** Correlation between histopathological parameters and stromal PDGFR-beta and tumoral VEGFR-2 expression in serous/ serous-papillary ovarian cancer

histopathological parameters	stromal PDGFR-beta expression	tumoral VEGFR-2 expression
FIGO pT	*p* = 0.150	*p* = 0.829
	*p* = 0.173	*p* = 0.001
pN	*p* = 0.674	*p* = 0.055
pM Residual disease after surgery histopathological grading	*p* = 0.144	*p* = 0.734
	*p* = 0.420	*p* = 0.820
	*p* = 0.003	*p* = 0.769

### High stromal PDGFR-beta expression correlates significantly with shorter overall survival in patients with high-grade serous ovarian cancer

For survival analyses, we focused on the subgroup of HGSOC samples (n = 205) as a homogenous patient collective with PDGFR-beta staining available for 199 samples. The median follow-up of the overall cohort was 34 months (range: 0 to 136 months). The cohort was divided into two groups according to the intensity of stromal PDGFR-beta staining. Weak to moderate staining was noted in 153 (76.9%) patients, strong staining in 46 (23.1%) patients.

No significant association between stromal PDGFR-beta expression and progression free survival was observed (data not shown). Patients with high stromal PDGFR-beta expression levels had significantly shorter overall survival rates compared to patients with low to moderate PDGFR-beta expression levels (Fig. 1D; p = 0.038; median overall survival of patients with weak/moderate PDGFR-beta: 69 months, 95% CI 47.3–90.4 months; median overall survival of patients with strong PDGFR-beta: 48 months, 95% CI 32.1–63.8 months).

In stratified survival analyses, no predictive value could be attributed to stromal PDGFR-beta expression. High PDGFR-beta levels showed significant correlation with shorter overall survival in a subgroup of patients treated with bevacizumab as maintenance therapy (n = 127, Fig. 1E; p = 0.037; median overall survival of 96 patients with weak/moderate PDGFR-beta: 75 months, 95% CI 64.7–85.3 months; median overall survival of 31 patients with strong PDGFR-beta: 52 months, 95% CI 38–66 months). However, the same trend could be observed in patients treated with platinum-based chemotherapy alone (n = 72) even though the results did not reach statistical significance in this smaller subgroup (Fig. 1F; p = 0.343; p = 0.343; median overall survival of 57 patients with weak/moderate PDGFR-beta: 50 months, 95% CI 22.3–77.7 months; median overall survival of 15 patients with strong PDGFR-beta: 32 months, 95% CI 13.6–50.4 months).

Further stratification, accounting for residual disease after surgery as strongest prognostic factor in ovarian cancer, showed that the prognostic value of stromal PDGFR-beta was confined to the subgroup of patients without residual disease after cytoreductive surgery. Thus, significant shorter overall survival was associated with high PDGFR-beta expression in patients who received bevacizumab and had no residual disease after surgery (Fig. 1G; p = 0.028; median overall survival of 51 patients with weak/moderate PDGFR-beta: 86 months, 95% CI 69–103 months; median overall survival of 10 patients with strong PDGFR-beta: 53 months, 95% CI 45.7–60.3 months). In contrast, stromal PDGFR-beta staining showed no impact on survival in patients with residual disease after surgery who received bevacizumab (Fig. 1H; p = 0.392; median overall survival of 17 patients with weak/moderate PDGFR-beta: 35 months, 95% CI 13.1–56.4 months).

When looking at the impact of both treatment regimens on patients after stratification into weak/moderate versus high PDGFR-beta expression, we observed that patients with weak to moderate PDGFR-beta expression significantly benefited from an anti-angiogenic therapy (p = 0.020, n = 153, supplementary Fig. 3A). However, the same trend was observed in patients with high PDGFR-beta expression levels, even though the difference in overall survival in this smaller subgroup did not reach statistical significance (p = 0.142, n = 46, supplementary Fig. 3B).

In the multivariate analysis including stromal PDGFR-beta intensity, therapy regiment and tumor rest after surgery, PDGFR-beta did not reach significance (p = 0.154, supplementary Table 1).

### VEGFR-2 expression analysis on tissue samples of ovarian cancer patients

**Fig. 2:**
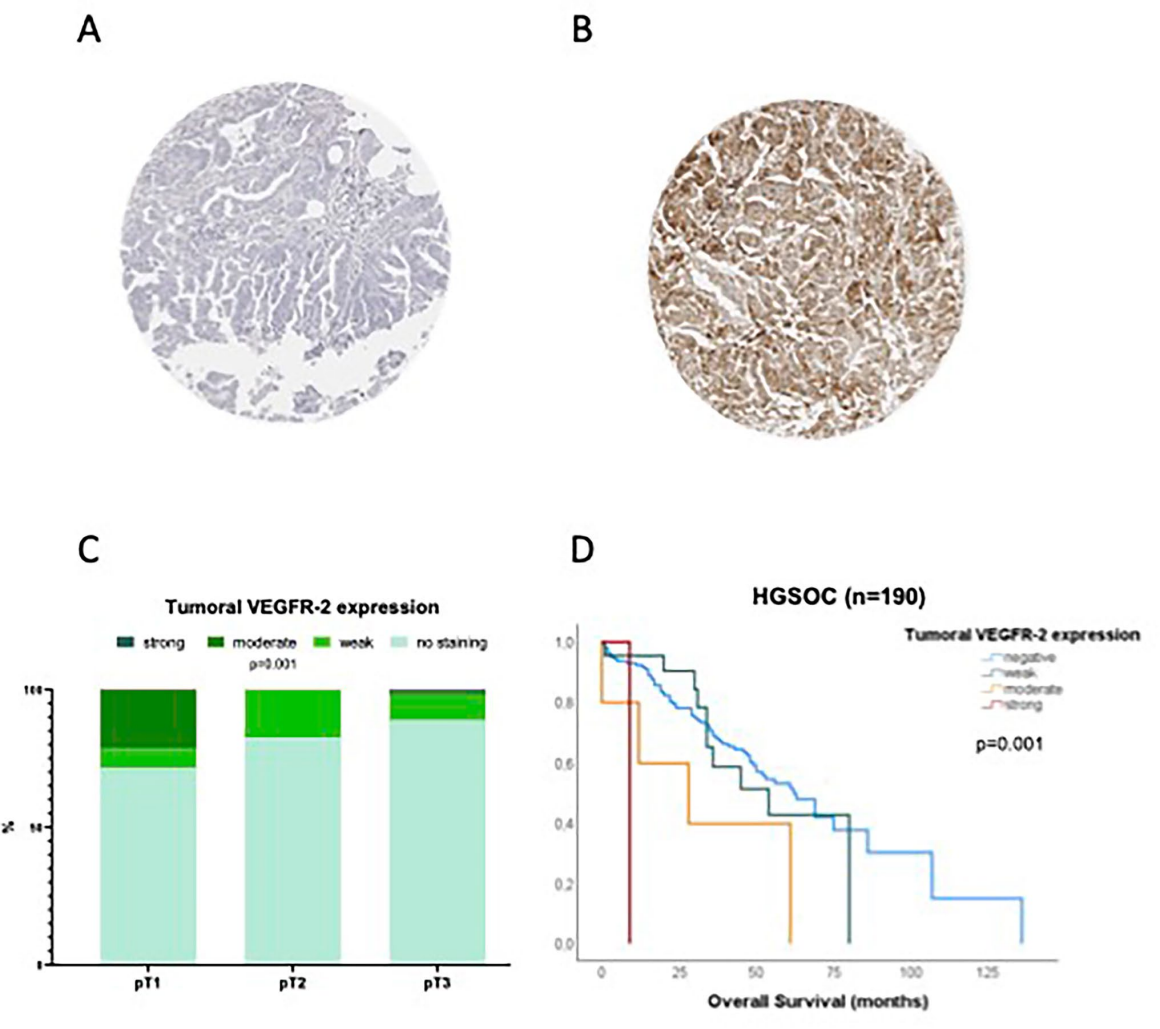
Tumoral VEGFR-2 expression. **A**–**B** VEGFR-2 immunostaining in serous ovarian carcinoma: **A** Absent VEGFR-2 staining. **B** Strong VEGFR-2 staining in the tumor. **C** Tumoral VEGFR-2 expression correlates significantly with pathological tumor stage (*p* = 0.001). **D** In high-grade serous ovarian cancer VEGFR-2 expression correlates significantly with overall survival (*p* = 0.001). Negative to weak staining was associated with longer overall survival

Representative images of tumors with varying staining intensity are shown in Fig. 2A–B. Tumor cell VEGFR-2 staining was seen in 33 (12.5%) tumor samples. Of these, staining was noted to be weak in 26 (9.8%), moderate in 6 (2.3%) and strong in 1 (0.4%) tumor sample. Most tumors (n = 209, 78.9%) lacked VEGFR-2 staining and 23 (8.6%) samples were not analyzable.

### Tumor cell VEGFR-2 expression in serous ovarian cancer and its association with clinical parameters

Statistical analyses revealed no significant association between tumor cell VEGFR-2 expression levels and nodal involvement, distant metastasis, FIGO stage, histopathological grading or postoperative residual tumor (Table 2). However, staining intensity correlated significantly with pathological tumor stage (pT, p = 0.001, Fig. 2C), where higher levels of VEGFR-2 were noted in early stage tumors.

In the subgroup of HGSOC (n = 190), we found a significant correlation between level of VEGFR-2 expression and overall survival rate (Fig. 2D; p = 0.0012; median overall survival of patients with negative VEGFR-2: 62 months, 95% CI 50–74 months; median overall survival of patients with weak VEGFR-2: 54 months, 95% CI 24.8–83.1 months; median overall survival of patients with moderate VEGFR-2: 28 months, 95% CI 0–62.3 months; median overall survival of patients with strong VEGFR-2: 9 months). Patients with negative (n = 162) or weak (n = 22) VEGFR-2 tumor cell expression levels showed significantly longer overall survival than those displaying a moderate (n = 5) or strong (n = 1) expression of VEGFR-2 in tumor cells. No significant association between VEGFR-2 expression and progression free survival was observed (data not shown).

## Discussion

Anti-angiogenic treatment with bevacizumab is commonly applied in advanced ovarian cancer. When applied as a maintenance therapy for 18 cycles in first-line therapy, improved progression free survival by approximately 4 months has been shown (Burger et al. 2011). However, application of therapy comes with potentially severe side effects, such as hypertension, wound healing disorder, gastrointestinal perforation and proteinuria (Li and Kroetz 2018; Wichelmann et al. 2021).

In recent decades, research has focused on identifying molecular markers that can predict response to therapy with bevacizumab (Schneider et al. 2012). In particular, molecular mediators of angiogenesis are at the center of attention as their levels of protein expression may predict response to anti-angiogenic treatment (Miyamoto et al. 2018). PDGFR-beta and VEGFR-2 are known to play a key role in angiogenesis and regulate vascular cell recruitment, the latter by binding to VEGF-A, the main target of bevacizumab (Ball et al. 2007).

As stated before, our previous work has identified high expression levels of angiogenesis related PDGFR-beta and VEGFR-2 as predictive biomarkers for primary resistance to platinum-based chemotherapy. It was assumed that these patients in particular might benefit from additional anti-vascular therapy, such as anti-VEGFR antibody therapy (Avril et al. 2017).

Therefore, in this study we evaluated PDGF-beta and VEGFR-2 expression in an extensive collective of 391 ovarian cancer samples included in a tissue microarray assay. We demonstrated that high stromal PDGFR-beta expression correlates with shorter overall survival, thus, revealing its potential as a prognostic marker in patients with high-grade serous ovarian cancer. Unfortunately, no predictive value for patients who were treated with bevacizumab could be ascribed.

PDGF is a mesenchymal protein that can be found in ovarian cells as it is embryologically derived from the Mullerian duct with mesenchymal components (Schmitt and Matei 2012). In ovarian tumors, its receptor PDGFR and especially the isoform PDGFR-beta is commonly expressed (Schmandt et al. 2003). The current study confirms this, and in contrast to previous work, our analyses additionally provide a detailed representation of protein localization, thereby highlighting the importance of the stromal PDGFR-beta protein fraction. In fact, stromal PDGFR-beta expression was predominantly detected, while expression in tumor cells was almost absent.

High levels of stromal PDGFR-beta expression significantly correlated with higher histopathological grading of serous and serous-papillary ovarian carcinomas. In line with previous studies, no further significant correlation with other clinical-pathological characteristics of patients was noted (Corvigno et al. 2016; Madsen et al. 2012).

PDGFR-beta is commonly expressed in HGSOC and as it is the most commonly diagnosed histopathological subtype, we focused our survival analyses on this subgroup of patients (Schmandt et al. 2003).

We have previously shown that increased PDGFR-beta expression correlates significantly with reduced overall survival in ovarian cancer patients (Avril et al. 2017). Moreover, Ferri-Borgogno et al. have also associated PDGFR-beta in cancer associated fibroblasts of the tumor microenvironment in HGSOC with unfavorable survival (Ferri-Borgogno et al. 2023). In accordance, we noted a significantly shorter overall survival for HGSOC patients with high stromal PDGFR-beta expression, stressing the potential of PDGFR-beta as a potential prognostic marker in HGSOC. In contrast to our previous work, analyzing the tumor samples by tissue microarray assay not only allowed us to differ between tumoral and stromal tissue compartments, it is also a method of expression analysis that can be applied more easily in everyday clinical practice compared to quantitative reverse-phase-protein-arrays, which was used before (Avril et al. 2017).

Unlike expected, stratified survival analyses showed no predictive value in the subgroup of patients that received therapy with bevacizumab. Independent of stromal PDGFR-beta expression levels, patients had a better prognosis when treated with bevacizumab. A significant difference was only noted in the subgroup of patients with low to moderate stromal PDGFR-beta levels, while analyses in the subgroup of patients with high stromal PDGFR-beta levels did not reach significance. This is likely due to the much smaller number of patients. Taken together, this suggests that stromal PDGFR-beta could serve as a prognostic marker for HGSOC patients but not as a predictive marker for therapy with bevacizumab. These observations applied in particular to patients without residual disease after cytoreductive surgery. When tumor tissue was not completely removed during surgery, stromal PDGF-beta expression levels did not predict the length of overall survival. This underlines the fact that residual tumor after surgery has the largest impact on prognosis in ovarian cancer while other modulating factors, such as PDGFR-beta expression play a subordinate role (Bryant et al. 2022).

In a similar study design, Corvigno et al. confirmed that a high PDGFR-beta positive stroma fraction correlates significantly with lower survival rates in serous ovarian cancer (Corvigno et al. 2016). They also examined perivascular staining and found an additional association between high perivascular staining and shorter overall survival in patients with intermediate to high grade tumors.

The VEGF-A/VEGFR-2 axis located on endothelial cells is considered the main pathway mediating angiogenesis in solid tumors and is a target of several anti-angiogenic agents. Of special interest is the expression of VEGFRs on tumor cells, suggesting an autocrine VEGF/VEGFR signaling (Masood et al. 2001). VEGFR-2 expression on ovarian cancer cells has previously been reported in small patient cohorts, although its functional significance is not completely understood (Abu-Jawdeh et al. 1996; Chen et al. 2004; Nishida et al. 2004). In contrast to these studies, we observed an almost absent VEGFR-2 protein expression on tumor cells in our tissue microarray assay. We assume that VEGFR-2 does not play a decisive role in the biology of the tumor cell—at least in advanced stages- but rather on endothelial cells during activation of tumor associated vessels and angiogenesis. In line with these findings, VEGFR-2 expression has previously been shown to have limited impact on response to treatment with bevacizumab (Nishida et al. 2004). Confirming these findings, our study showed that no or weak tumoral VEGFR-2 expression correlates with longer overall survival compared to higher levels of protein expression, independent of the anti-angiogenetic therapy regiment.

It should also be considered that our findings are based on tissue microarray analyses and that this method may have limitations that need to be taken into account. (I) There may be limited sensitivity, especially affecting low expression levels, (II) the heterogeneity of the tumor cannot be captured and (III), blood vessels can only be analyzed to a limited extent with this method.

For both proteins, no significant correlation with progression free survival could be observed. This contrasts with the effect of bevacizumab therapy improving progression free survival but not overall survival (Burger et al. 2011). Still, in a retrospective study design as ours, detecting the progression of cancer depends on symptom burden and is therefore determined by the timing of patients consulting their doctors. In addition, even though the majority of patients were treated at our hospital, a large number continued their treatment at local hospitals where the protocol of screening to determine progression might be different. In contrast, overall survival is a unambiguous outcome measure and therefore a more reliable endpoint.

When identifying molecular markers that can predict response to therapy with bevacizumab, angiogenic molecules are at the center of attention. High levels of expression are expected to be predictive of better response to anti-angiogenic treatment (Miyamoto et al. 2018). Not only do PDGFR-beta and VEGFR-2 both play a key role in angiogenesis, but the latter also regulates vascular cell recruitment when binding with VEGF-A, the main target of bevacizumab (Ball et al. 2007). Nevertheless, in our findings, high expression levels were not associated with better outcome in HGSOC patients treated with bevacizumab. This surprising result has been described before. Published data suggests, that the expression level of VEGF in tissue and plasma is not predictive for response to bevacizumab in colorectal cancer patients (Pohl et al. 2011; Vlajnic et al. 2011). Also, patients with metastatic breast cancer showing a VEGF-A gene amplification have no benefit from treatment with bevacizumab (Schneider et al. 2013). While underlying mechanisms remain unclear, researchers assume that through amplification, VEGF-A can no longer be blocked successfully and that high levels of angiogenic determinant might lead to inhibition of bevacizumab (Miyamoto et al. 2018; Schneider et al. 2013).

Once more, it becomes obvious that the biology of angiogenesis is a very complex mechanism involving different pathways and a variety of regulating proteins. Accordingly, the role of PDGFR-beta and VEGFR-2 and their clinical prognostic value may not directly be linked to the treatment with bevacizumab (Schneider et al. 2008). The expression of PDGFR-beta in the stromal compartment underlines the importance of the tumor microenvironment.

Schmitt et al. describe a destabilizing effect of PDGFR inhibitors on pericytes through which a local enrichment of chemotherapy is promoted in tumor tissue (Schmitt and Matei 2012). In our study no separate investigation of protein expression in pericytes was carried out, representing a limiting factor for this study. Further studies with focus on the stromal microenvironment including the role of pericytes are needed to explore the effects of anti-angiogenetic therapy.

Further limiting the value of this study is the retrospective analysis on a limited number of tumor samples (n = 199), making prospective studies for validation necessary. Another limitation of our study is the potential bias in patient selection. Patients receiving bevacizumab therapy are generally in better clinical condition and have a better overall prognosis compared to those who do not receive anti-angiogenic therapy due to severe comorbidities. However, it is important to note that bevacizumab is primarily administered to patients with homologous recombination (HR) proficient tumors. These tumors are characterized by intact DNA-repair mechanisms, which contribute to resistance against DNA-damaging therapies such as platinum-based chemotherapy (Stiegeler et al. 2024). Consequently, this subgroup is associated with worse clinical outcomes and higher rates of chemoresistance. This highlights the complexity of interpreting outcomes in patients treated with bevacizumab, as their better initial health may counterbalance the adverse prognosis linked to HR proficiency.

The tumor microenvironment of serous ovarian cancer remains at focus of investigations and reliable biomarkers that predict response to anti-angiogenic therapy in ovarian cancer patients still need to be identified (Monk et al. 2016; Han et al. 2010).

In summary, our results align with many previous studies. No predictive value of PDGFR-beta nor VEGFR-2 expression for therapy with bevacizumab could be ascribed. However, we demonstrate that high stromal PDGFR-beta expression is associated with adverse outcome and therefore might serve as a prognostic marker in high-grade serous ovarian cancer.

## Supplementary Information

Below is the link to the electronic supplementary material.

Supplementary file1 (PDF 795 KB)

## Data Availability

The data that support the findings of this study are not openly available due to reasons of sensitivity and are available from the corresponding author upon reasonable request.
