# Supplementary material for: Stromal PDGFR-beta expression is a prognostic factor in high-grade serous ovarian cancer patients but is it also predictive for response to antiangiogenic treatment?
Source: J Cancer Res Clin Oncol. 2025 Jan 24;151(2):44. doi: 10.1007/s00432-025-06090-4 (PMC11759463; doi:10.1007/s00432-025-06090-4)
Supplement: Supplementary file 1 — Supplementary file1 (PDF 795 KB) [file 432_2025_6090_MOESM1_ESM.pdf]

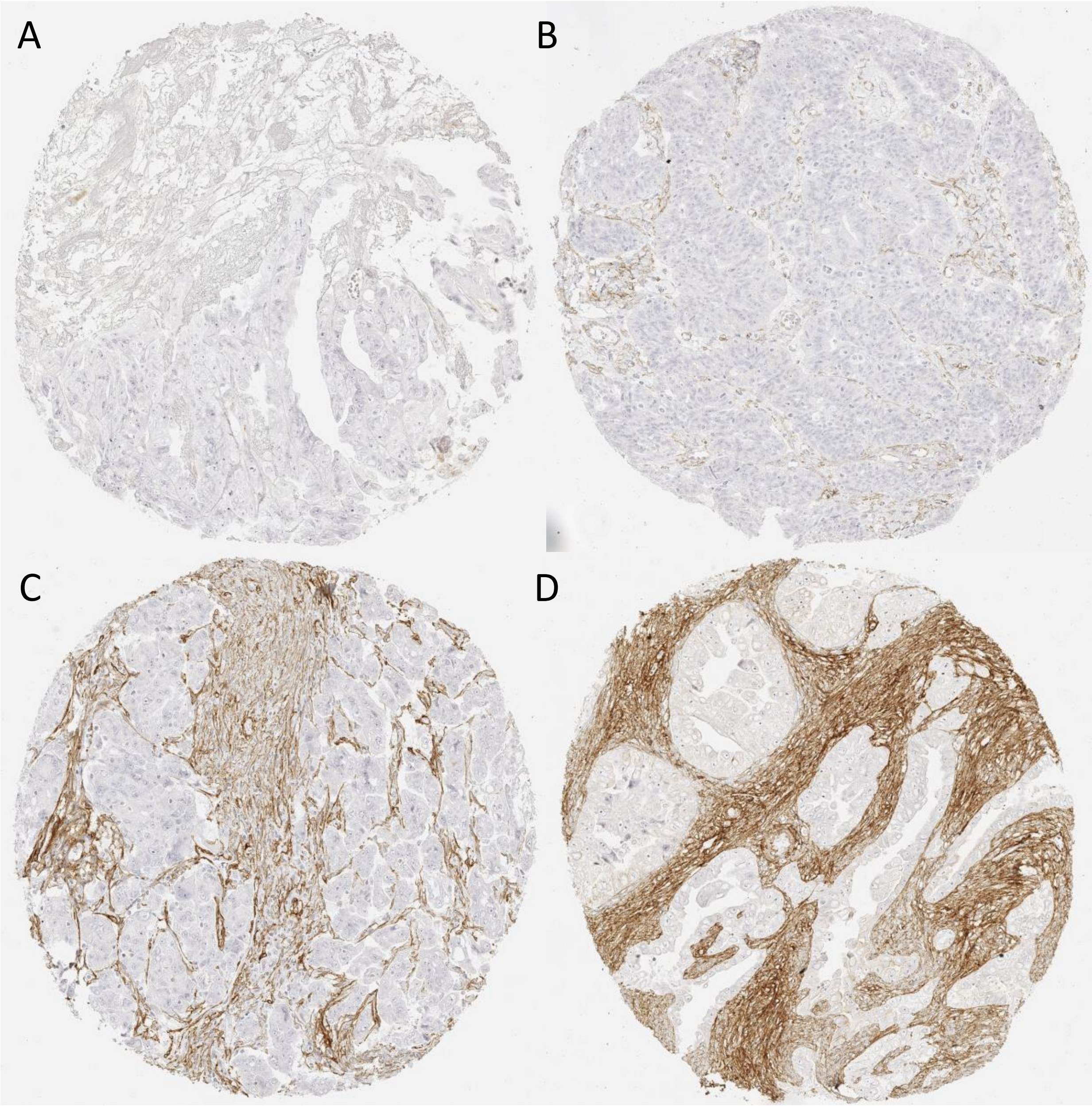

Supplementary Figure 1: PDGFR-beta immunohistochemistry staining scores in HGSOC. Staining scores ranged from 0 to 3. (A) Score 0 indicates complete absence of detectable staining (negative). (B) Score 1 indicates that the staining is clearly visible but of weak intensity. (C) Score 2 indicates moderate staining not reaching the intensity of score 3. (D) Score 3 is defined as intense dark brownish staining of cell membranes and the cytoplasm (strong).

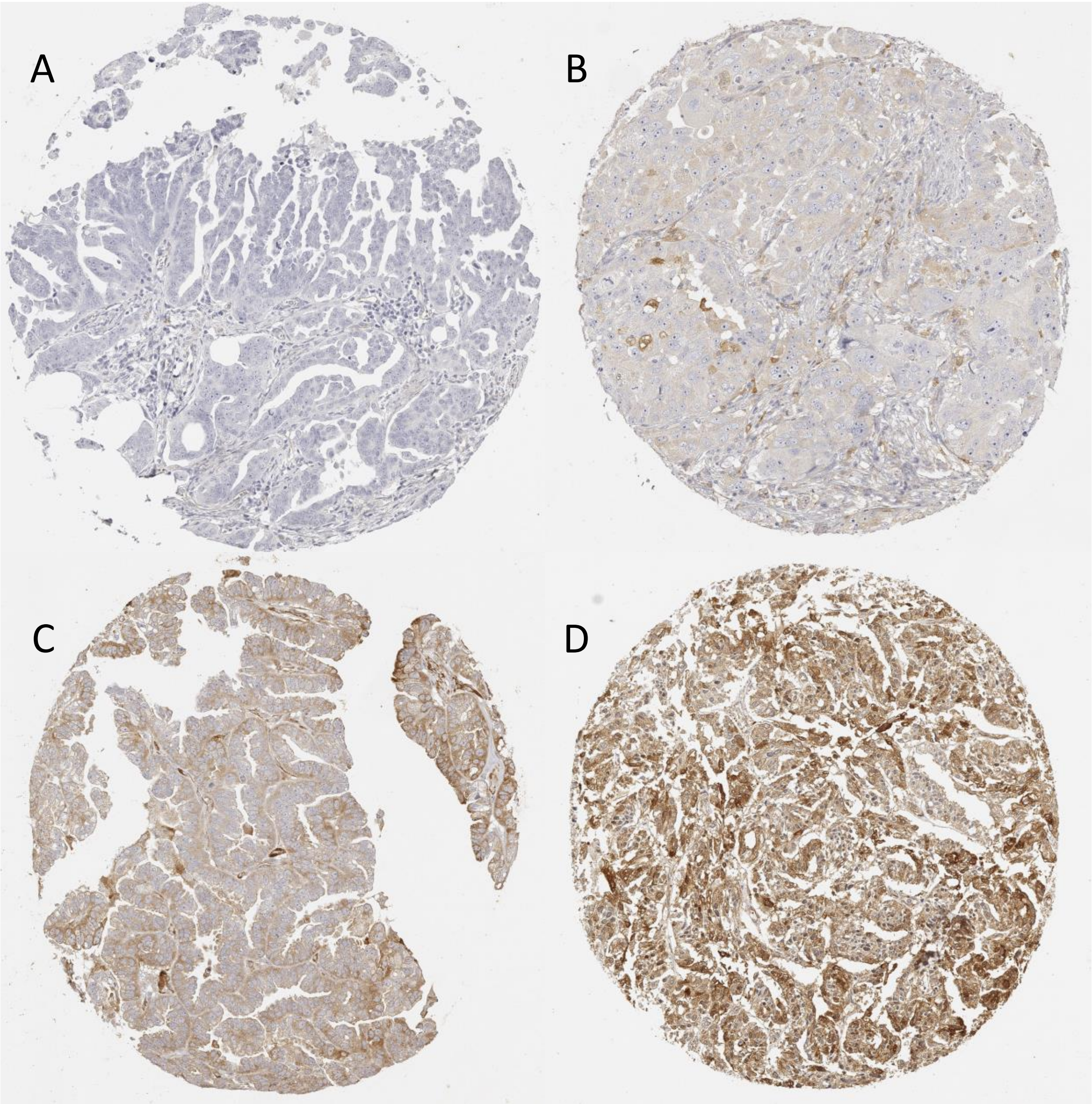

Supplementary Figure 2: VEGFR-2 immunohistochemistry staining scores in HGSOC. Staining scores ranged from 0 to 3. (A) Score 0 indicates complete absence of detectable staining (negative). (B) Score 1 indicates that the staining is clearly visible but of weak intensity. (C) Score 2 indicates moderate staining not reaching the intensity of score 3. (D) Score 3 is defined as intense dark brownish staining of cell membranes and the cytoplasm (strong).

A

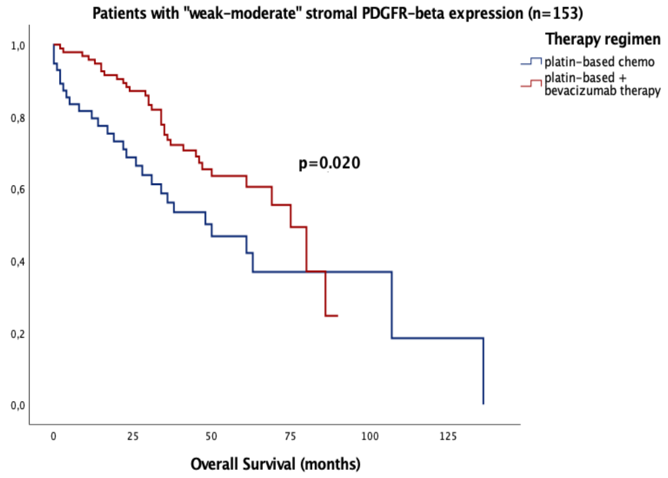

B

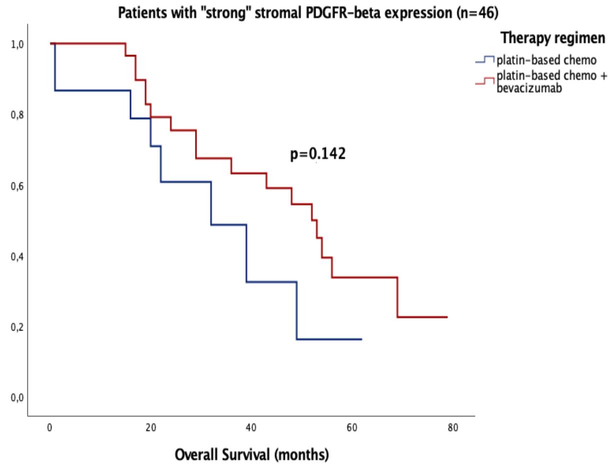

Supplementary Figure 3: PDGFR-beta expression in HGSOC treated with bevacizumab. (A) Patients with weak to moderate PDGFR-beta expression had significantly longer overall survival when receiving bevacizumab (p=0.020; median overall survival of 57 patients treated with platin-based chemo: 50 months, 95% CI: 22.3 – 77.6 months; median overall survival of 96 patients treated with platin-based chemo and bevacizumab: 75 months, 95% CI: 64.7 –85.3 months). (B) In patients with high PDGFR-beta expression levels no significant difference in overall survival was observed between the two treatment regimens (p=0.142; median overall survival of 15 patients treated with platin-based chemo: 32 months, 95% CI: 13.5 – 50.4 months; median overall survival of 31 patients treated with platin-based chemo and bevacizumab: 52 months, 95% CI: 38 – 66 months).

		Overall survival		
	**categories**	**p-value**	**HR[1]**	**95%CI**
Stromal PDGFR-beta	weak+moderate vs. strong	0.154	1.532	0.852-2.756
Macroscopic tumor rest after surgery	no vs. yes	< 0.001	1.943	1.381-2.733
Bevacizumab Therapy	no vs. yes	0.041	0.572	0.335-0.978

[1], hazard ratios adjusted for all other variables

Supplementary Table 1: multivariate analysis including stromal PDGFR-beta intensity, therapy regime and tumor rest after surgery.
